# Barriers and Facilitators to Mental Health Service Integration: A Scoping Review

**DOI:** 10.1111/inm.13449

**Published:** 2024-10-09

**Authors:** Adam Searby, Dianna Burr, Renae Carolin, Alison Hutchinson

**Affiliations:** ^1^ Monash University School of Nursing and Midwifery Melbourne Australia; ^2^ Centre for Quality and Patient Safety Research, School of Nursing and Midwifery Deakin University Geelong Australia; ^3^ Change to Improve Mental Health (CHiME) Translational Research Partnership Deakin University Geelong Australia

**Keywords:** mental health, mental health nursing, primary health care, psychiatric nursing, service integration, systems integration

## Abstract

Mental health service integration currently has no consensus definition and exists in a variety of settings, including primary care, addiction treatment and chronic disease management, and mental health nurses have often experienced efforts at service integration with varying degrees of success. The intent of mental health service integration is to enable collaboration between mental health services and other healthcare providers to improve service access and the care provided to individuals with mental health issues or mental illness. This scoping review aimed to explore service integration between mental health services and with a specific focus on those evaluated in peer‐reviewed, primary literature, to determine facilitators and barriers to service integration. Using the Arksey and O'Malley's framework for scoping reviews, we located 3148 studies, with screening narrowing final papers for inclusion to 18. Facilitators to service integration included clinician education, adequate resourcing and an interdisciplinary approach, while barriers included staff factors such as a reluctance to work with individuals with mental illness, consumer level barriers such as poor mental health literacy, ‘territorialism’ among staff and organisational climate. Research indicates that service integration is an effective means to detect and treat mental health issues in settings that do not traditionally provide mental health care, lowering the costs of providing healthcare and providing improved care for mental health needs; however, there are several barriers to be addressed to achieve full implementation of integration models.

## 1 Introduction

As a concept, mental health service integration has no consensus definition; literature refers to integration between mental health services and primary care or addiction treatment services, with little reference as to how a mental health service can integrate into the overall healthcare landscape (Peterson 2013). Integrated care has been defined by Hetrick et al. (2017) as a way to ‘join up’ mental and physical healthcare, alongside social support services, to achieve holistic healthcare that is coordinated and organised around the needs of the individual healthcare consumer. A systematic review of youth mental health service integration conducted by Kinchin et al. (2016) found that service integration was often defined in four broad contexts: (1) collaboration across sectors, with these sectors including those that exist beyond mental health treatment, such as the education and justice sectors; (2) improved coordination of care within the healthcare sector; (3) improved family coordination; and (4) the notion of ‘patient‐centred integration’, with a specific focus on the wellbeing of the consumer.

In 2003, The World Health Organization (WHO, 2003) recommended that mental health services be integrated into general health services to reduce the stigma associated with help‐seeking for people with mental illness, overcome potential shortages of skilled mental health professionals, encourage early identification of mental illness and as a strategy for mental healthcare to reach underserved populations. Despite these recommendations, in many global jurisdictions service integration has not been successfully realised. For example, a recently conducted Royal Commission into the mental health system in the state of Victoria, Australia (2021) recommended that services should ‘Establish a responsive and integrated mental health and wellbeing system, in which people receive most services locally and in the community throughout Victoria, close to their families, carers, supporters and networks’ (p. 39). The report describes a complex, fragmented mental health system that lacks integration to the detriment of providing a holistic, coordinated approach as described by Hetrick et al. (2017).

Existing literature mainly explores integration from the primary health perspective, with individuals attending primary health care, and with a focus on prevention and early intervention in deteriorating mental health (Jemimah et al. 2018; Miller et al. 2014; Moscrop, Siskind, and Stevens 2011). However, consumers of mental health services have substantially poorer physical health than the general population; individuals with schizophrenia have a shorter life expectancy than the general population (Marder et al. 2004), have higher rates of obesity, cardiovascular disease and diabetes, and poor dietary habits and rates of physical inactivity (Firth et al. 2019). Furthermore, smoking rates for individuals with mental illness are significantly higher than those with no mental illness diagnosis (Lasser et al. 2000; Lawrence, Mitrou, and Zubrick 2009). These findings indicate an urgent need to also view service integration from the perspective of the mental health service, particularly to provide service linkage to wider healthcare services.

Much of the research evaluating the success of integration, both in respect of clinical outcomes and healthcare consumer satisfaction, has been conducted in the United States Veteran's Health Administration. Usually, these evaluations involve individuals seeking primary healthcare and testing whether integration achieves an increase in treatment uptake for mental illness and alcohol and/or substance use disorders (Johnson‐Lawrence et al. 2012). Analysis of care episodes contained within the Nationwide Patient Care Database found that this approach increased encounters of mental health care within the Veteran's Affairs patient cohort, also showing an increase in second encounters of care for mental illness (Wray et al. 2012). However, the results indicated that these programmes of integration were more effective for high‐prevalence mental health disorders such as depression, anxiety and alcohol abuse, with less success reported for low‐prevalence disorders such as schizophrenia and bipolar affective disorder, and alcohol dependence.

Consumers have also been found to be satisfied by service integration. For example, a study of consumers being discharged from a hospital in Canada (Forchuk et al. 2021) had them describe the mental health service as providing reassurance when transitioning from hospital to the community, helping to reduce stigma, increasing social connectedness, enhancing continuity of care and recovery and reducing feelings of isolation. Criticisms of the programme included a sense that the peer supporters were ‘intruders’, communication issues were evident and more in‐person support was desired, although consumers interviewed for this study were positive about improved integration between mental health services and community providers. However, this was a single‐site qualitative study; little evidence exists exploring consumer acceptability of integrated services, or the ideal composition of integrated services to meet consumer needs.

The literature exploring clinician and care provider perspectives on integrated services is also scant. Integration between mental health settings and primary care settings was reported as highly valued among 59 staff working in these settings in Los Angeles, USA; however, this survey study indicated that integration and enhanced communication were more highly valued by primary care staff than mental health staff (Urada et al. 2012). An Australian study of alcohol and other drug treatment nurses (*n* = 46) found substantial negative perceptions of service integration, such as a perception that mental health services were engaging in a ‘take over’ of alcohol and other drug treatment services, stigma towards people who use alcohol and other drugs predominated in mental health treatment settings, and mental health services had a different perception of risk (Searby et al. 2022).

### 1.1 Aim

The aim of this scoping review was to explore existing examples of service integration between mental health services and other healthcare services, with a specific focus on those evaluated (either their clinical outcomes, healthcare consumer or clinician experiences) in peer reviewed, primary literature, to determine facilitators and barriers to service integration.

## 2 Methods

Scoping reviews are typically used to explore the ‘conceptual boundaries’ of a topic (Peters et al. 2015). Given the diverse nature of studies exploring service integration models, in addition to the various methods (or lack) of evaluation, a scoping review methodology was chosen. Scoping reviews are also not limited by the quality of the literature, and given the diversity of study methodologies, we determined this as the best method to gain an overview of mental health service integration (Davis, Drey, and Gould 2009). This scoping review is reported in accordance with the PRISMA Extension for Scoping Reviews (PRISMA‐ScR): Checklist and Explanation reporting guidelines (Tricco et al. 2018).

### 2.1 Identification of Review Aims

We aimed to explore existing models of mental health service integration. To gain a wide overview of published studies that both describe and evaluate service integration, we did not limit our search to mental health and alcohol and other drugs (AOD) service integration, rather we conducted a broad search for instances where a mental health service had been integrated with others (for instance, primary care). Our secondary aim was to define the strengths and limitations of these models, including common barriers and facilitators to successful mental health service integration.

### 2.2 Identification of Relevant Studies

The eligibility criteria for the search strategy for this scoping review are defined in Table 1. Searches of CINAHL, PubMed, MEDLINE and APA PsycInfo were conducted in September 2023, using combinations of the search term ‘mental health’ and ‘service integration’. The search term was deliberately kept broad to encompass research exploring the integration of mental health services with all other services, such as primary care, community care and alcohol and other drug treatment services.

**TABLE 1:** Review inclusion and exclusion criteria.

Inclusion criteria	Exclusion criteria
Peer‐reviewed, academic journal articles or grey literature	Theses/dissertations or non‐peer‐reviewed articles
Published from 1990 to include historical models of integration	Published before 1990
Published in English	Published in languages other than English
Primary research of mental health service integration with evaluation discussed in academic paper or academic papers describing mental health service integration with other services	Mental health service not involved in integration model
Where papers involved primary research all study designs were included	Discussion paper, including those providing a discussion of perceptions of ‘ideal’ service integration
	Editorials, letters, commentaries or opinion papers

### 2.3 Plotting the Data

Data were extracted from included papers using the tool shown in Table 2. Two members of the research team (Author 1 and Author 2) extracted the data. All members of the research team discussed and agreed on the extracted data and key study findings.

**TABLE 2:** Characteristics of selected studies.

Author, (year)	Study setting	Study aim	Integration components	Target population	Description of integration	Evaluated?	Evaluation method	Significant outcomes
Abraham et al. (1991)	Older adult neuropsychiatry clinic, Virginia, USA	To describe a model of outpatient older adult nursing services focussed on the community integration of patients and families	Mental health services Physical health services Neuropsychology Community care	Older adults with neuropsychiatric and behavioural concerns	Multidisciplinary clinic model, incorporating mental health, neuropsychiatric and physical health clinicians	No		Integration created several lines of referral to the clinic. Creates ‘wraparound’ service for traditionally underserved population. Clinic used as a teaching facility for medical and nursing staff, emphasising a holistic approach to neuropsychiatric and behavioural concerns in older adults. Focus on integration of community resources into care. Accessibility noted to be limited due to restrictions on physical opening hours.
Anastas et al. (2019)	Behavioural Health Home (BHH), Oregon, USA	To describe early stages of integrating medical care teams in mental health and addiction treatment settings	Mental health services Alcohol and/or other drug treatment services Primary care	Adults with serious mental illness (SMI) and substance use disorders (SUD)	Integration of primary care into SMI and SUD treatment (behavioural health services) in ‘health homes’ for Medicaid recipients	Yes	Qualitative evaluation: focus groups to determine agency definitions of the BHH model, leadership interviews to determine strengths and weaknesses of the BHH model	Several barriers and facilitators identified among clinical staff, including motivation to work with the BHH population and a lack of preparation and education in SMIs and SUDs. Staff also had a poor understanding of integration and did not view it as part of their job. Not enough patients of the BHH wanted or needed integration services, and the ‘fast‐paced’ primary care scheduling did not fit the needs of the SMI/SUD patient cohort. No extra reimbursement for the BHH model, despite extra services and staff time provided to individuals with SMI/SUD. A need for cultural change in workforce development and clinic workflows was identified as a need for integration to succeed.
Ayalon et al. (2007)	Primary care clinic, San Francisco, USA	To compare two models of service delivery: an enhanced referral model versus an integrated mental health, substance use and primary health service among ethnic minority older adults	Primary care Mental health services Alcohol and/or other drug treatment services	Older adults seeking treatment from primary care clinics, particularly ethnic minorities including Black Americans	Enhanced referral: Mental health and substance use treatment services provided separately from primary care services, with referral taking place within 2–4 weeks of primary care appointment. Integrated model: Co‐location of mental health and substance use treatment services with primary care clinics	Yes	Quantitative evaluation: Randomisation of 155 eligible consumers (56% Black, diagnosed with depression (82%), anxiety (32%) and/or problematic alcohol use (22%)) to enhanced referral arm (*n* = 82) or integrated treatment arm (*n* = 73)	Black consumers in the integrated treatment arm were more significantly more likely to have at least one mental health or substance use treatment visit. Time between assessment and first mental health or substance use treatment visit was significantly shorter in the integrated treatment arm. The authors postulate that an integrated model provides better access to ethnic minorities, in this case Black elderly Americans, in part due to co‐location of services and familiarity of staff and trust in clinicians.
Barraclough, Longman, and Barclay (2016)	Rural primary healthcare service, New South Wales, Australia	To describe a nurse‐practitioner‐led primary healthcare rural mental health service, and to explore how this service integrated into other services in the community	Primary care (Nurse Practitioner) Mental health services	Clients presenting to a non‐government organisation (NGO) located in a regional New South Wales town centre. Consumers mostly adults, however some in the 10–14‐year age bracket (2%)	Embedded full‐time, mental health qualified nurse practitioner offering flexible, immediate short‐ and long‐term support to clients with both mental ill health and dual diagnosis; this service included ‘street‐based’ outreach from the NGO clinic	Yes	Descriptive case study methodology: multiple sources of quantitative and qualitative data used (including interviews with 21 stakeholders)	Integration was described as going beyond delivering combined services in substance use treatment and mental health, linking community and welfare services into community treatment. Strong support for nurse practitioner model from community services, including benefits to staff: as a referral point, and a guide to practice. Stakeholders described existing silos in health service structures, with co‐location of the nurse practitioner improving collaboration. Geographical distance between NGO service and inpatient mental health service was noted to lead to poor awareness of the nurse practitioner by inpatient mental health staff.
Chiodo et al. (2022)	Youth services network, Ontario, Canada	To describe the implementation of an integrated youth service in a small urban city and rural county in Canada	Mental health services Alcohol and/or other drug treatment services Community‐based youth organisations	Youth aged 12–26	Integrated youth service hubs based in Ontario communities, providing access to mental health, substance use, community, and social support services (such as education, employment, and housing services) delivered in ‘youth friendly’ spaces	Yes	Qualitative: ‘developmental approach’, incorporating key informant interviews (*n* = 12) of site supervisors and programme managers	Collaboration between network partners was felt to reduce ‘silo mentality’, with a shared understanding of barriers to integration. Successful integration often relied on a motivated service director acting as an ‘individual driver’ of service collaboration and integration. Concern among key informants that stigma is a barrier to participation at rural sites. Concerns raised on the sustainability of the funding model for the project.
Druss et al. (2017)	Behavioural Health Homes, USA	To compare quality and outcomes of care between a behavioural health home (BHH) and usual care	Mental health services Primary care	Adults with serious mental illness (schizophrenia, schizoaffective disorder, bipolar disorder, major depression, obsessive‐compulsive disorder or posttraumatic stress disorder, with or without comorbid substance use)	Integration between community mental health service and Federally Qualified Health Centres, safety net clinics that provide primary care to poor and underserved populations in the BHH model. Integration aims to reduce morbidity and poor‐quality medical care for individuals with serious mental illness	Yes	Quantitative: randomised control trial. 447 patients with serious mental illness and one or more cardiometabolic risk factors randomised to BHH (*n* = 224) or usual care (*n* = 223) for 12 months	Individuals in the BHH model received significantly higher quality care than those enrolled in usual care. The BHH had no significant effect on most clinical outcomes, including cardiometabolic parameters. The authors note that both study groups demonstrated significant improvement in cardiometabolic outcomes between baseline and 12‐month follow‐up, potentially due to screening and community referral in the usual care group. The authors recommend future research into programmes combining medical care and lifestyle factors, due to high rates of smoking, poor diet, low physical activity, and obesity in this cohort.
Edward, Hearity, and Felstead (2012)	Alcohol and other drug treatment service, Victoria, Australia	To illustrate, using four case studies, how dual diagnosis (comorbid mental ill health and alcohol and/or other drug use) treatment has been approached	Mental health services Alcohol and/or other drug treatment services	Individuals seeking treatment from non‐government organisation (NGO) alcohol and/or other drug treatment services	Building the capacity of NGO alcohol and other drug treatment service clinicians to respond to mental ill health, building collaboration, cooperation and cross‐referral between alcohol and other drug treatment services and mental health services	No		The authors highlight that individuals with dual diagnosis have higher mortality rates, with mental illness and substance misuse leading to several barriers to receiving appropriate health care. The authors indicate that when dual diagnosis is systematically identified and responded to in a timely manner, there are benefits to service consumers. A cultural shift is needed at organisational level, particularly regarding policy and funding agreements, that reduce segregated, standalone services in preference of fully integrated dual diagnosis services.
Elphinston et al. (2021)	Emergency department, Queensland, Australia	To describe and examine a new specialist alcohol and other drug brief intervention team integrated into the emergency department, particularly regarding the identification of individuals at risk of AOD‐related harm	Alcohol and/or other drug treatment services Emergency departments	Individuals over 12 years old presenting to the emergency department of a 436‐bed public hospital in Brisbane.	Integration of an AOD service into the emergency department, to provide opportunistic AOD screening and brief intervention using a harm reduction approach	Yes	Quantitative: interrupted time series analysis combined with cost‐outcome analysis	Period post implementation (May–December 2016) saw 1025 referrals to the service; median age of individuals referred was 38, with 62% male. The cost per referral was $420 during this period. Team integration was stated to overcome barriers to integrating brief interventions into practice, such as time‐poor staff or low engagement in providing screening and brief intervention. Integrated team members also provided staff education and support, which was hypothesised to increase ED staff capacity to refer individuals with AOD use.
Gutmanis et al. (2017)	Older adult neuropsychiatry clinic, Ontario, Canada	To describe the application of a Collective Impact framework to the design, implementation, and ongoing development of an integrated service in Ontario	Primary care Community health services Mental health services	Older adults with neuropsychiatric symptoms associated with mental health and addiction issues	Creation of ‘geriatric cooperatives’: integrating subregion geriatric service providers to build on local capacity and develop plans to address system gaps	No		Success with the integration model was attributed to the involvement of ‘key players’ in services, in addition to leadership and funding commitments. Some difficulties in developing common terminology between services. ‘Drift’ from the integration mandate was reported, with adherence difficult in the light of evolving agency mandates. Fatigue described among clinicians with ‘constant change’.
Hansson et al. (2012)	Mental health service, Stockholm, Sweden	To describe the formulation of a health and social care ‘consortium’ in Sweden that had developed a shared treatment model between mental health services and social services for people with mental ill health	Public health social services Psychiatric clinic	Individuals with mental ill health	Project leaders from both departments were engaged to develop a coordination plan to government to secure funding for initial planning to develop new premises integrating the services—longitudinal project	Yes	Qualitative case study approach: interviews with key informants (*n* = 12), an exploration of programme documents, and structured interviews with case managers	The advantages, disadvantages, and challenges of a longitudinal and contextualised case study method for understanding how coordination and improvements to care are provided for clients with complex needs. Where full structural integration is not possible, then client‐level coordination roles in each sector are useful when they also work closely together across sectors for shared clients. A final lesson for current policy, based on the micro level outcomes, is to advance the work towards fully integrated care in a bottom‐up approach by the inclusion of macro management levels via shared steering group for representatives from both services.
Lawn et al. (2014)	Primary health service (general practitioner), Adelaide, South Australia	To present a complete system of health care to clients, integrating existing primary health care services and community mental health services with General Practice and non‐government services. To support increased teaching, training, and education opportunities for health professionals.	Community mental health Primary healthcare Mental health (adult and youth) Dentistry Allied health Pathology Youth services Sexual health Drug and alcohol counselling Chronic disease management	Individuals seeking primary care through a general practitioner (GP)	A purpose‐built GP Plus site was used to introduce a Community Mental Health Centre, with a ‘hoteling’ model used to house staff from diverse services on site	Yes	Ethnographic methodology of focus groups, day‐to‐day interactions with staff, and observations within the centre recorded as reflections by the researcher officer	Infrastructural impediments to collaboration; each team retained a separate system of governance, the nature of the physical space impeded collaboration, with services spread across floors and behind locked doors, and diverse information management systems were a barrier to sharing individual medical records. Territorialism: shared spaces were ‘claimed’ by teams labelling desks; however, this was overcome when collaborative care was required for complex clients. Interprofessional practice (IPP) simply not on the agenda for clinicians. IPP was accepted by management; however, the change process was not implemented sustainably. Resistance to changing practice was reported. A suggested solution was to incorporate university training where future health professionals train in an interdisciplinary, collaborative manner.
Maar et al. (2009)	Aboriginal mental health service, Ontario, Canada	To study the strategies, strengths and challenges related to collaborative Aboriginal mental health care	First nations community health Mental health	Canadian First nation's adults	Over the past decade First Nations (Indigenous communities) in the Manitoulin District in Northern Ontario, Canada have created an integrated community‐based mental health services system by successfully pooling resources and developing collaborative programmes	Yes	Participatory action research. Ethnographic Interviews and Focus Groups with Service Providers (*n* = 31) The key informant interviews were conducted with the Knaw Chi Ge Win team, visiting mental health consultants, therapists and social workers in the mainstream service system, and tribal police. Focus group participants included First Nations community administrators and community‐based health workers.	Formal opportunities to share information, shared protocols and ongoing education support this model of collaborative care. To function well in interprofessional teams, healthcare professionals must overcome barriers such as professional rivalry and negative stereotypes and thus require specialised training. Positive outcomes associated with this model include improved quality of care, cultural safety, and integration of traditional Aboriginal healing with clinical approaches. Ongoing challenges include chronic lack of resources, health information and the still cursory understanding of Aboriginal healing and outcomes.
Oviedo et al. (2023)	Community mental health service, Maryland, USA	To demonstrate that integration of substance use disorder treatment into outpatient community mental health care is feasible and beneficial	Outpatient community mental health services Substance use disorder treatment service	Adult population seen in the Catholic Charities of Baltimore community mental health clinics	Integrating substance use disorder treatment into existing community mental health service in response to the United States opioid epidemic	No		An expectation was made that all therapists in the clinic were expected to screen for, diagnose and treat substance use disorder. A barrier to integration was the need to develop partnership development with local substance use treatment services. Many individuals with substance use disorder only presenting at the clinic for treatment, meaning clinic needed to obtain licence to treat substance use disorder alone.
Perkins et al. (2006)	Rural mental health service, New South Wales, Australia	To establish and document approaches to integrating private psychiatrist and public mental health services	Community mental health teams (CMHTs) General practitioners (GP) Consultant psychiatrists from public and private sectors	Residents in a remote area of NSW with the highest level of socioeconomic disadvantage in Australia	Existing Community mental health teams (CMHTs), general practitioners (GP) and other agencies were provided with clinical and broader support services by visiting consultant psychiatrists from public and private sectors. The occasions of service were logged, audited and relevant provider groups were interviewed	Yes	Mixed methods: services were audited, and relevant provider groups were interviewed	Key to the model was the balance of primary (advance planned specialist visits) and secondary activities (mentoring, case review, secondary consultation). Increased resources and flexibility in their use, such as access to psychiatrists and mental health teams, enabled improved access to mental health services in the remote context. Specialist case review, mentorship and secondary consultation were undertaken by visiting psychiatrists to improve capability among general practitioners, hospital staff, Aboriginal health service staff and other agencies; the best evidence of the value of secondary activity was the strong relationship between visiting psychiatrists and community mental health teams.
Peterson et al. (2016)	Chronic care service, South Africa	To develop a district mental healthcare plan (MHCP) in South Africa that integrates mental healthcare for depression, alcohol use disorders and schizophrenia into chronic care	Chronic disease management Mental health Alcohol and other drug treatment	Adults with multiple chronic diseases, including HIV and non‐communicable diseases	Phase 1: Collaborative care packages were developed following a situational analysis, theory of change workshops and formative interviews Phase 2: Community health worker outreach team training programme to identify and refer people with mental health disorders and trace disengaged chronic health care patients	Yes	Mixed methods: Participants included primary healthcare nurses (*n* = 4), lay counsellors (*n* = 4), auxiliary social workers (*n* = 2), patients who received counselling for depression (*n* = 6), patients who attended psychosocial rehabilitation groups (*n* = 6) and caregivers of patients attending psychosocial rehabilitation groups (*n* = 4).	Advantages of incorporating mental health into existing service delivery platforms noted as opportunity to provide holistic care, reduce stigma, and leverage existing resources to promote efficiency and effectiveness. Limitations noted with staff were a lack of confidence by nurses to diagnose mental health issues, and low confidence of counsellors to follow up patients with alcohol and other drug issues; this was noted to be considered a social problem requiring referral to a social worker. Low mental health literacy and psychiatric stigma were noted among patients using services, requiring education and provision of information; this extended to alcohol consumption, where there was a noted defensiveness to divulging consumption levels. Structural and organisational challenges that impeded identification and/or referral of depression or alcohol use disorders by nurses included high patient loads and space constraints that limited consultation.
Reifels et al. (2018)	Indigenous primary care services, Australia	To improve Indigenous access to culturally appropriate mental healthcare services	Primary care Mental health services	Indigenous Australians with high prevalence mental ill health (such as depression)	Access to Allied Psychological Services (ATAPS) programme and suicide prevention programme available through 31 regional primary health networks, with enhanced referral process to ATAPS for general practitioners. Suicide prevention programme enabled enhanced referral for those deemed at risk of suicide by general practitioners, hospital wards, acute mental health teams, Indigenous health organisations, emergency departments or drug and alcohol services	Yes	Qualitative (stakeholders) (*n* = 31)	Study findings highlight that concerted national attempts to enhance mainstream primary mental healthcare for Indigenous people are critically dependent on effective local agency‐ and provider‐level strategies to optimise the integration, adaptation, and broader utility of these services within local Indigenous community and healthcare service contexts. Despite the explicit provider focus, this study was limited by a lack of Indigenous stakeholder perspectives. There is a requirement for treatment‐coordinating referrers exist, have close agency collaboration with Indigenous health services and where experienced professionals are engaged with Indigenous communities.
Reiss‐Brennan (2006)	Primary care service, Utah, USA	To describe the implementation of an evidence‐based, integrated quality improvement mental health programme in a primary care setting	Mental health service Primary care	Adult Americans attending primary care clinics for chronic conditions such as diabetes and asthma	Development of a mental health treatment cascade that recognises more complex comorbidities risk level for mental ill health rises. As the risk category rises, additional mental health services are engaged. The cascade was designed to recognise the burden that clinicians are under managing multiple clinical issues, providing a supported mechanism to identify and treat mental health conditions	Yes	Cost–benefit analysis	Trend analysis, comparing primary care clinics with those using the mental health treatment cascade, shows significantly better rates of detection of depression in those using the mental healthcare cascade. Expected increases in claims costs for depression were experienced when the mental health care cascade was implemented, and prescription claims increased as individuals were diagnosed, provided a prescription, and filled the medication from the pharmacy. Overall, results show the mental health cascade reducing the burden on primary care physicians, improving clinical outcomes, increasing depression detection rates, improving healthcare claims costs and improving patient satisfaction.
Yu et al. (2016)	Sexual health clinic, New York, USA	To analyse data from a pilot programme that aims to link public health and substance abuse treatment services	Alcohol and other drug treatment Sexual health clinics	Adults (over 18 years of age) in New York City accessing public sexual health clinics for sexually transmitted disease treatment	Screening, Brief Intervention and Referral to Treatment (SBIRT) services were implemented and provided in three sexual health clinics across a 4‐year period, with trained clinicians providing brief intervention and referral for those assessed as having a substance use disorder	Yes	Pre‐ and post‐test	Over 146 000 clinic patients screened over a five‐year period, with 74% adherence to screening indicates that the implementation of SBIRT is feasible in a clinic setting. 39% of patients who received a positive screen for at‐risk substance use did not receive intervention due to staff limitations and ‘heavy patient flow.’ Only 19% of patients who received a referral for substance use disorder connected with substance use treatment services; the authors hypothesise that for patients with sexually transmitted diseases, particularly HIV, substance use treatment is a secondary concern, and the lack of co‐located treatment services was also an issue. Supported referral was considered a way to address low substance use disorder take‐up.
Zivin et al. (2010)	Department of Veteran's Affairs, USA	To assess the prevalence of diagnosed mental health conditions among primary care patient populations in association with PC‐MHI programmes, overall and for patient subpopulations that may be less likely to receive mental health treatment	Mental health services Primary care settings	Veterans receiving primary care services	Integration of mental health services into Veterans Affairs primary health services; these included co‐located collaborative care and case management	Yes	Difference‐in‐differences analysis	When compared to non‐integrated clinics, integrated clinics increased diagnoses of depression, anxiety and post‐traumatic stress disorder. Integration increased detection of mental health disorders for both genders, however, did not increase the detection of alcohol use disorders among women. Key limitation is that analysis was carried out at a facility level rather than a patient level. There may have been facility characteristics that influenced changes in the rates of diagnosis.

### 2.4 Collating, Summarising and Reporting the Outcomes

The final stage of the scoping review process (Arksey and O'Malley 2005) is to collate, summarise and report the findings of the literature selected during the review process. Of the 18 papers describing the integration of mental health services with other services, 10 were from the United States, six were from Australia, three were from Canada and one each was from Sweden and South Africa. Eleven papers explored integration between mental health and primary care, five between mental health and alcohol and other drug treatment services, two focussed on older adult services, two explored the integration of First Nations (Aboriginal) health services and one specifically focussed on youth service. In terms of research design, authors of seven papers used a quantitative methodology, six qualitative, three mixed‐methods and five did not evaluate the model of integration; these authors only provided a description of the integration model.

## 3 Results

**FIGURE 1:**
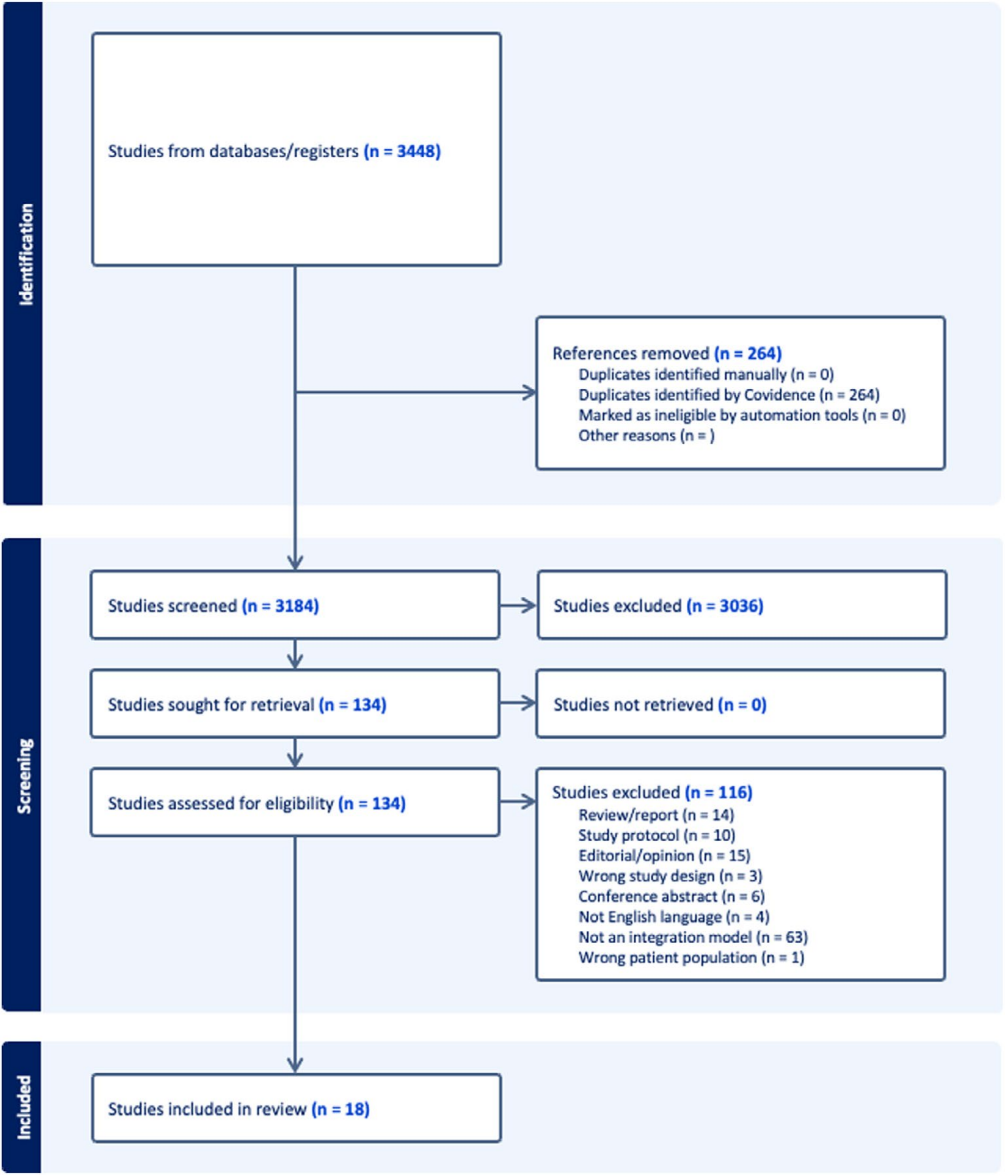
PRISMA diagram of search flow.

Searching the electronic databases returned a total of 3448 studies, and after duplicates were removed, 3148 studies remained. Title and abstract screening were conducted by two authors (Author 1 and Author 2), with papers included if they explored a model of service integration between mental health and any other service/s. After title and abstract screening, 136 studies the remaining references progressed to full text screening, with each paper requiring the approval of the two authors for inclusion in the final review. The final number of included papers was 21. Figure 1 shows the PRISMA diagram for the study (Liberati et al. 2009).

Five key areas emerged from the literature. The first was descriptions of service integration between mental health and other healthcare services, the second was evaluation of whether service integration improves outcomes for consumers of mental health and other services, the third was identification of facilitators of service integration and the final theme was exploration of barriers to service integration.

### 3.1 Descriptions of Service Integration Between Mental Health and Other Healthcare Services

All papers included in this review described the use of a range of methods of service integration with the common goal of improving access, inclusion and effectiveness of mental health services among other healthcare services and providers. Ten of these involved integration with primary care services (Anastas et al. 2019; Ayalon et al. 2007; Barraclough, Longman, and Barclay 2016; Druss et al. 2017; Gutmanis et al. 2017; Lawn et al. 2014; Perkins et al. 2006; Reiss‐Brennan 2006; Zivin et al. 2010). Predominantly, these implementation efforts aimed to ‘embed’ mental health services into primary care settings to improve screening, diagnosis and rapid acceptance and referral to treatment, if needed. In most cases, this was achieved by having either enhanced referral mechanisms in place or specialised mental health clinicians accessible in public health settings. However, in one rural Australian setting a mental health nurse practitioner was used to drive integration and provide care continuity for individuals with mental illness (Barraclough, Longman, and Barclay 2016).

Three papers exploring mental health service integration in primary care settings also had a specific alcohol and other drug treatment focus (Anastas et al. 2019; Ayalon et al. 2007; Lawn et al. 2014), with an aim to implement universal screening and linkages to brief intervention or treatment. Three papers specifically focused on the integration of mental health and alcohol and other drug treatment services (Chiodo et al. 2022; Edward, Hearity, and Felstead 2012; Oviedo et al. 2023), and one described the integration of an alcohol and other drug service into an emergency department to provide screening and brief intervention to individuals recognised as being at risk of alcohol and other drug‐related harm (Elphinston et al. 2021).

Other integration efforts described in papers in this review included one that integrated mental health services into services that catered for older adults with neuropsychiatric and behavioural concerns (Abraham et al. 1991), one that integrated social services with mental health services to improve access (Hansson, Øvretveit, and Brommels 2012) and integration of a service providing chronic disease management with mental health and alcohol and other drug treatment (Peterson et al. 2016). In another model, mental health services were integrated into a sexual health clinic due to an increased incidence of alcohol and other drug use in this population (Yu et al. 2016), while the remaining two models integrated mental health care into services for First Nations (Aboriginal) individuals (Maar et al. 2009; Reifels et al. 2018).

### 3.2 Evaluation of Whether Service Integration Improves Outcomes for Consumers of Mental Health and Other Services

Of the 19 papers included in this review, 14 reported evaluation outcomes of service integration. Engagement in treatment was investigated by two papers: Ayalon et al. (2007) randomised 155 eligible healthcare consumers with depression, anxiety or alcohol abuse to an enhanced referral arm (*n* = 82) or an integrated treatment arm (*n* = 73). The researchers found that those in the integrated treatment arm had greater engagement and significantly shorter time between initial assessment and mental health or alcohol and other drug treatment visits, with integrated services providing better access to minority groups due to colocation of services and trust in clinicians. A similar evaluation methodology was utilised by Druss et al. (2017), who randomised 447 adults with serious mental illness to an integrated Behavioural Health Home model (*n* = 224) or usual care (*n* = 223) for 12 months. Their results showed that individuals randomised to the integrated Behavioural Health Home model received significantly higher quality care; however, no significant difference was found between the clinical outcomes of both groups, including cardiometabolic parameters.

Yu et al. (2016) used a pre‐ and post‐test methodology to analyse data from a pilot integration programme in a sexual health clinic, where over 146 000 clinic patients were screened over a 5‐year period. They found a high level of adherence to screening by clinicians (74%), a low uptake of referrals to alcohol and other drug treatment services (19%), and several patients of the service who received a positive screen for substance use disorder did not receive an intervention due to ‘heavy patient flow’ in the clinic (39%). In relation to diagnosis, Zivin et al.'s (2010) difference‐in‐differences analysis of an integrated Veteran's Affairs clinic showed that compared to non‐integrated clinics, integrated clinics were associated with increased diagnoses of depression, anxiety and post‐traumatic stress disorders. However, the integrated clinics were not associated with increased detection of alcohol use disorders among women.

An interrupted time series analysis combined with cost–benefit analysis was undertaken to determine the efficacy of the specialist alcohol and other drug brief intervention team integrated into a Queensland emergency department (Elphinston et al. 2021). The researchers concluded that referral uptake after implementation of the service was successful, with 1025 referrals to the service in the period May–December 2016. The cost of each referral was noted to be $420, and although the study did not focus on clinical outcomes, the authors showed successful, cost‐effective uptake of the implementation model. A similar cost–benefit evaluation was conducted by Reiss‐Brennan (2006) of the integration of a mental health programme into a primary care setting, finding that significantly better rates of depression detection occurred using the integration model; however, there were increases in claims costs for treatment and prescriptions after diagnosis.

### 3.3 Identification of Facilitators to Service Integration

Several papers included in this review described factors that facilitated successful integration. There were three dominant facilitators of integration: education, interdisciplinary approaches and resourcing.

#### 3.3.1 Education

Facilitators of integration typically related to culture change, where integration was deemed to be normal practice rather than an exception, and usually this required staff buy‐in through education. Using an older adult neuropsychiatry clinic as the focus of the integration, Abraham et al. (1991) developed a teaching facility for medical and nursing staff where an integrated, holistic approach to care was modelled for students during their placement. Similarly, in their participatory action research study of the integration of a First Nations (Aboriginal) health service, Maar et al. (2009) found that participants described ongoing education as essential to enhance cultural understanding of both clinical and Aboriginal approaches to healing and to support professional development in this specialised area of integrated care.

Secondary consultation activities, such as those used by Perkins et al. (2006), involved mentoring, interdisciplinary case review and secondary consultation to build capacity in the workforce and provide ongoing education to existing community mental health teams and primary care general practitioners. The authors argued that the best evidence of the value of these activities was a strong relationship between visiting psychiatrists and the community teams that were the focus of the study. In other studies, those responsible for integration provided ongoing education to increase referral and recognition capability; for example, Elphinston et al. (2021) used education to increase the capability of emergency department staff to respond to alcohol and other drug use, resulting in a higher rate of successful referrals to the integrated team.

#### 3.3.2 Interdisciplinary Approach

The ability to work within interdisciplinary teams and provide an interdisciplinary approach was frequently mentioned as a facilitator to successful mental health service integration. In the literature, this was described as being aided by formal opportunities to share information and protocols (Maar et al. 2009). Likewise, a strong interdisciplinary relationship between integration specialists (visiting psychiatrists) and the community mental health team was highlighted as a facilitator of service integration (Perkins et al. 2006). As outlined in the next section, the lack of an interdisciplinary approach was frequently described as a barrier to effective service integration. An intervention to address the lack of interdisciplinary collaboration and perceived ‘siloing’ of services, where significant resistance to interdisciplinary practice was encountered, was based on university training involving interdisciplinary and collaborative communication (Lawn et al. 2014).

#### 3.3.3 Resourcing

Where programmes were well‐resourced, both financially and with personnel, successful mental health service integration was often achieved. Often, this resourcing was led by service leadership; for example, Chiodo et al. (2022) described a service leader who was committed and passionate about the integration project, which translated to resourcing the project and continued to attract funding for sustainability. Similar findings were highlighted by Gutmanis et al. (2017), who indicated that resourcing by engaging ‘key players’ to implement funding commitments was essential for the success of their programme integrating older adult mental health and primary care services.

Perkins et al. (2006) also recognised the need for adequate resourcing in their Australian rural mental health integration model, where an increase in resources in respect of integration components such as specialised mental health teams and clinicians was recognised as essential to achieving integration. Further, flexibility in these increased resources, for instance changing the time allocated by visiting clinicians and teams, was considered essential in the rural context, particularly when individuals preferred local access to clinicians rather than travelling for specialist care.

### 3.4 Exploration of Barriers to Service Integration

Although facilitators were mentioned in some of the papers examined for this review, barriers to integration were overwhelmingly described. These barriers were commonly described as substantial impediments to ongoing sustainability of the mental health service integration model, and although some of the authors described methods to overcome these barriers, largely, encountered barriers impacted care delivery in each model. Four categories of barriers were described: staff factors, consumer level barriers, staff ‘territorialism’ and organisational climate.

#### 3.4.1 Staff Factors

Staff factors were mentioned in five of the papers included in this review. These factors are mostly related to the confidence and competence of clinical staff to include the assessment and management of either mental illness or alcohol and/or other drug use in their practice. For example, Anastas et al. (2019) noted that clinical staff did not understand integration, did not view integration as part of their role or had insufficient education or experience in working with complex serious mental illness or substance use disorder, leading to higher rates of staff burnout and turnover. Time pressures were mentioned in several papers as reasons for not completing integration tasks. For instance, in Elphinston et al.'s (2021) study of integrated care for people at risk of harm from alcohol use in an emergency department in Queensland, Australia, time pressures and low engagement in service integration were both noted as reasons provided for not completing screening and brief intervention tasks required to identify and address risky alcohol use.

Low self‐confidence in the ability of nurses to recognise and refer common mental health issues, and the low confidence of counsellors to follow up individuals referred to them for mental illness were identified as substantive barriers to integration (Petersen et al. 2016). These barriers resulted in low referral rates, requiring a need for role clarification and enhanced training for clinicians. A failure to refer patients who received a positive screen for risky substance use to treatment services was attributable to high workloads and an increased flow of individuals through the clinic environment, meaning referral follow‐up was often sacrificed to complete clinic tasks (Yu et al. 2016).

#### 3.4.2 Consumer‐Level Barriers

In addition to staff factors being barriers to mental health service integration, there were also consumer‐level barriers that impeded the integration process. In Peterson et al.'s (2016) study, poor mental health literacy was a significant barrier to seeking and accepting help for mental health challenges, also resulting in a ‘defensiveness’ when individuals were screened for alcohol consumption. Stigma was also present and considered a barrier to help‐seeking and integration. Other consumer‐level factors act as barriers to integration, such as those identified in Yu et al.'s (2016) study, where only 19% of individuals who received a referral to substance abuse services actually attended the service for treatment. In this case, the authors hypothesised that substance use was a secondary concern for individuals living with sexually transmitted diseases, such as HIV. These findings were reinforced by those of Anastas et al. (2019), where individuals seeking care from a Behavioural Health Home model did not perceive a need for mental health services, and the ‘fast‐paced’ nature of the primary healthcare model that was the focus of the integration did not suit the needs of those with serious mental illness.

#### 3.4.3 Staff ‘Territorialism’

‘Territorialism’, defined by Lawn et al. (2014) as territorial behaviour observed by clinicians using shared spaces, was considered a significant barrier in a number of studies included in this review. In this case, territorialism was described as ‘claiming’ of shared workspaces with staff placing personal items and labels on these spaces, leading to a perception of ownership of shared space by a team. Territorialism was described as contributing to the ongoing ‘siloing’ of care, where services are separated physically, with different administrative structures, and often had an accompanying attitude among clinicians that certain tasks or diagnoses were outside their remit (Black et al. 2020). Territorialism was noted to be a substantive barrier to the stated objectives of service providers working in a collaborative fashion, and even extended to the reservation of tables in shared meal room spaces (Lawn et al. 2014).

The authors of three included papers specifically mentioned the concept of siloing, with integration of services designed to address this issue. For example, Barraclough, Longman, and Barclay (2016) noted the existence of an existing siloed model, with a nurse practitioner co‐located with services to improve collaboration. Chiodo et al. (2022) also described an existing siloed care model that needed to be challenged by network partners in the integration model with clear, shared understanding of the barriers to implementation. Edward, Hearity, and Felstead (2012) also encountered siloing of services in their study of alcohol and other drug treatment service in Victoria, Australia, noting that a ‘cultural shift’ was needed at an organisational level, especially regarding policy and funding agreements, that reduce segregated services.

#### 3.4.4 Organisational Climate

Successful service integration was described as requiring a favourable organisational climate; ‘key players’ from services need to be involved and provide a strong commitment to the leadership of integration models for those working clinically to maintain a similar commitment to the successful implementation of integrated services. There is a risk of ‘drift’ from the mandate guiding service implementation, particularly as this mandate evolved within agencies. This was reported in the study as a key barrier to maintaining adherence to integration models (Gutmanis et al. 2017). As previously mentioned, the organisational climate required strong leadership to focus on the tendency of staff to be reluctant to work with individuals with serious mental illness, also viewing integration as not part of their role (Anastas et al. 2019). These findings were supported by Chiodo et al. (2022) in their Canadian study of youth services integration, where success in integrating services was attributed to strong organisational leadership from a motivated service director who was noted to work as an individual driver of service collaboration.

An organisational culture shift was also identified by Edward, Hearity, and Felstead (2012) as a requirement for successful integration, particularly the formulation of policy and funding agreements that reduce the tendency for services to become siloed and standalone. However, in some instances, where changes were accepted by management, these did not translate to clinician‐level practice. For example, Lawn et al. (2014) found that the change process regarding interprofessional practice to improve service integration was not implemented sustainably, resulting in substantial resistance to practice change. A lack of resources to support service integration was mentioned in several papers. For example, Peterson et al. (2016) described a lack of physical resources, such as space to consult with individuals using the integrated service and to collaborate with clinicians, while Yu et al. (2016) found that the high workload of clinicians once the integration model was implemented resulted in a lack of follow through of referrals made to substance use services.

## 4 Discussion

This scoping review aimed to review current examples of mental health service integration to explore the components of these models, and barriers and facilitators to achieving integration. Although there is evidence to indicate that mental health service integration can result in better patient outcomes (Crowley and Kirschner 2015; Donald, Dower, and Kavanagh 2005; Hetrick et al. 2017), a consensus on what is meant by service integration is sorely needed. The diversity of integration models in this review shows that mental health service integration can take several forms, often attempting to integrate better mental health care into existing systems, such as primary care or sexual health services. In the case of alcohol and other drug use, integration can mean incorporating screening, referral and brief intervention methods or embedding specialist clinical teams into places where individuals who use alcohol and/or other drugs frequently present. An example of where this strategy was employed was the Elphinston et al.'s (2021) study of a Queensland emergency department, which saw an increase in referral rates; however, the study did not explore the take‐up of these referrals, highlighting a need for longitudinal observation of individuals who receive integrated service interventions to determine whether they are successful in achieving their stated aims.

Many of the barriers identified in this scoping review were driven by health system factors, including the organisation of health service structures and funding mechanisms. Several studies included in our review identified funding commitments as a challenge to maintaining integration models, and in a healthcare environment where resourcing is challenging, funding for mental health initiatives may not be a priority when considering other funding needs. These concerns were echoed in a systematic review conducted by Wakida et al. (2018), where ongoing funding for integration initiatives was recognised as a challenge for sustainable service integration in several studies. However, research indicates that these issues may be ameliorated by effective stakeholder engagement (Andrea et al. 2023). Mental health nurses have a role to play in this area, as advocates for both individual consumers and healthcare systems that have implemented mental health treatment, and to establish collaborative relationships with clinicians outside traditional mental health settings (Shell et al. 2019).

The studies included in this review also indicate that staff factors are an issue. If staff are hesitant, or even disinterested, in providing care to people with mental illness, the sustainability of the integration model is severely threatened. Previous research has illustrated the importance of the attitude and willingness of clinicians to work with people with mental illness, such as the work of Björkman, Angelman, and Jönsson (2008), which found that this may be diagnosis dependent; the results of a survey of 120 nurses showed that schizophrenia was the diagnostic group that nurses do not wish to work with due to a perception of danger and unpredictability. However, research indicates that this barrier can be modified with education. In a randomised control trial of medical students in Canada, Papish et al. (2013) found significant reductions in stigma and increases in confidence in working with individuals with mental illness for those who received an educational intervention, and this is another key role for mental health nurses working within integrated services by providing support to interdisciplinary colleagues and advocating for the mental health needs of consumers.

A variety of efforts at incorporating mental health or alcohol and other drug treatment into settings where it has not traditionally been incorporated are included in this review. However, none of these models comprise a fully integrated mental health system that operates across all healthcare services. The examples of service integration included in this review have been driven by a need to provide care for people with mental illness or a deterioration in their mental health; however, it is apparent that mental health needs to be the focus of an ‘all of system’ approach. This concept is outlined by the American Academy of Paediatrics, who describe a task force that introduced integration across an entire paediatric children's hospital to address high rates of perinatal mood and anxiety disorders, which are recognised as causing poor maternal, child and family outcomes (Jarvis et al. 2021). In this instance, ‘buy‐in’ was created at a high level, indicating the need for a strong leadership culture to ensure sustainability of integration interventions.

Strong leadership is required to ensure that services communicate and collaborate without resorting to treating healthcare consumers and clinical issues in isolation, and this was evident in studies included in this review; most notably the study by Chiodo et al. (2022) where an individual leader was considered a key driver of the success the service integration model. Further, inadequate resourcing was considered a significant barrier to the success of integration, ranging from staff and training to physical resources such as appropriate workspaces that facilitated integration. Along with strong leadership being required to champion and facilitate implementation of an integrated system, cultural change is needed at all levels of the system to overcome the ‘siloed service’ mentality that is in direct opposition to service integration efforts. Aside from facilitating successful integration, poor resourcing has been implicated in burnout and intention to leave the mental health specialty among Australian clinicians (Scanlan and Still 2019). There is a role for mental health nurses to play in successful service integration, both as drivers of change and advocates for individuals in need of mental health care.

### 4.1 Strengths and Limitations

To our knowledge, this review is one of the few to explore existing service integration models in terms of their barriers and facilitators; however, there are limitations that should be considered. Our review only included literature published in peer‐reviewed, academic journals; description and evaluation of service integration models may exist in local health service documents and therefore have not been included using our methodology. We considered this method essential to ensure that evaluation literature had undergone a peer‐review process, and although diverse methodologies were used across the studies included in this scoping review, this method ensured a level of consistency in the quality of the included papers. However, in accordance with established literature on the conduct of scoping reviews, we did not conduct quality assessments of the included papers.

### 4.2 Relevance for Clinical Practice

This scoping review highlights several implications for mental health nurses. Our review has highlighted several service‐level factors; however, further research is needed to explore clinician attitudes to service integration, particularly in settings where there is fear, reluctance or hesitancy to implement components of integration models, such as mental health screening or brief intervention (such as brief interventions used for alcohol use). In most settings where service integration occurs, mental health nurses have a role to build capacity in staff who may have a lack of training or knowledge on working with individuals with mental illness, as well as to advocate for the inclusion of mental healthcare in all settings. In this context, implementation science should be used to inform the design of integration strategies, ensure the fidelity of integration strategies and models, and to ensure the sustainability of service changes, with particular attention given to involving mental health nurses in the design of service integration. Policy concerning service integration should involve mental health nursing input, and consider the need for strong leadership, cultural change and adequate resourcing to ensure the success of integration models.

## 5 Conclusion

Mental health service integration has been shown in the literature to be an effective means to detect and treat mental ill health in settings across the healthcare spectrum, lowering the costs of providing healthcare and providing better care to individuals who experience mental ill health. Service integration has also been shown to be effective for problematic alcohol and other drug use. However, several barriers to successful service integration exist, including poor resourcing, a lack of sustainable funding, and clinician reluctance to implement integration components. Facilitators that address these issues include strong leadership commitment to service integration and education on mental ill health for clinicians who have not traditionally worked in this area.

## Author Contributions

Each author certifies that their contribution to this work meets the standards of the International Committee of Medical Journal Editors.

## Conflicts of Interest

The authors declare no conflicts of interest.

## Data Availability

The authors have nothing to report.
